# Is hip fracture surgery safe for patients on antiplatelet drugs and is it necessary to delay surgery? A systematic review and meta-analysis

**DOI:** 10.1186/s13018-020-01624-7

**Published:** 2020-03-12

**Authors:** Zhanyu Yang, Jiangdong Ni, Ze Long, Letian Kuang, Yongquan Gao, Shibin Tao

**Affiliations:** Department of Orthopaedics, The Second Xiangya Hospital, Central South University, No. 139 Renmin Street, Changsha, Hunan 410000 People’s Republic of China

**Keywords:** Hip fractures, Meta-analysis, Platelet aggregation inhibitors, Aspirin

## Abstract

**Background:**

Hip fractures are common and account for a large proportion of orthopedic surgical admissions in elderly patients. However, determining the timing for surgery has been controversial for patients who develop hip fractures while on antiplatelet treatment.

**Methods:**

Computerized databases for studies published from the inception date to January 2020, including the Cochrane Library, PubMed (Medline), EMBASE, Web of Science^TM^, ClinicalTrials, ClinicalKey, and Google Scholar, were searched using the keywords “Hip AND Fracture”, “Antiplatelet”, “Antithrombocyte”, “Platelet aggregation inhibitors”, “Aspirin”, “Plavix”, and “Clopidogrel”.

**Results:**

In total, 2328 initial articles were identified. Twenty-four studies with 5423 participants were ultimately included in our analysis. Early surgery was associated with an increased transfusion rate in the antiplatelet group compared to the non-antiplatelet group (OR = 1.21; 95% CI, 1.01 to 1.44; *p* = 0.03). Early surgery for hip fracture patients on antiplatelet therapy was associated with a greater decrease in hemoglobin compared to delayed surgery (WMD = 0.75; 95% CI, 0.50 to 1.00; *p* < 0.001). However, early surgery appeared to decrease the length of hospitalization (WMD = − 6.05; 95% CI, − 7.06 to − 5.04; *p* < 0.001) and mortality (OR = 0.43; 95% CI, 0.23 to 0.79; *p* = 0.006).

**Conclusion:**

It is unnecessary to delay surgery to restore platelet function when patients with hip fractures receive antiplatelet therapy. Furthermore, early surgery can significantly reduce mortality and hospital stay, which is conducive to patient recovery. Future randomized trials should determine whether the results are sustained over time.

## Background

Hip fractures are among the main causes of orthopedic surgical admissions and life-threatening injuries that occur worldwide, mainly in elderly individuals. Older patients are particularly vulnerable to sustaining hip fractures because of the high prevalence of osteoporosis or osteopenia. According to the American Academy of Orthopaedic Surgeons, each year, the number of hospital admissions due to hip fractures can reach up to 350,000 [1]. By the year 2040, there will be more than 500,000 people with hip fractures per year, with an annual medical expenditure of at least $9.8 billion [1, 2]. Although the development of surgical instruments and medical technology for early mobilization has advanced significantly, the mortality rate remains high. The cause of this high mortality rate is not entirely clear, and most of the deaths are ascribed to comorbidities, including cardiovascular disease [3, 4]. Antiplatelet drugs are simultaneously used in most hip fracture patients for primary and secondary prevention of cardiovascular or cerebrovascular events. Because the inhibitory action of drugs on platelets is irreversible and mature platelets do not synthesize new proteins, antiplatelet drugs make platelets ineffective for approximately 7 days, the mean lifetime of a platelet [5].

Despite the obvious advantages in the prevention and treatment of cardiovascular diseases, the continued use of antiplatelet drugs perioperatively may have great risks. Clopidogrel therapy in cardiac surgery without preoperative disruption increased hemorrhagic risks, transfusion demands, and infection with a poor prognosis [6]. The potential hematoma risk in orthopedic surgery [7] forces the withdrawal of antiplatelet drugs and delays surgery for at least 5 days to allow platelet function to return to an adequate status. Currently, no agreed upon guidelines exist for the appropriate surgical time for patients suffering hip fractures while on antiplatelet therapy, and there is a marked divergence of opinion on the final results of early and delayed surgical intervention [8].

Therefore, the purpose of this review was to identify whether early surgical intervention can be safely implemented on patients who develop hip fractures while on antiplatelet therapy to promote satisfactory outcomes. A secondary aim was to determine whether early or delayed surgery was more appropriate for those patients. Moreover, we attempt to establish a framework for managing hip fracture patients with antiplatelet therapy.

## Methods

### Literature search

This review was performed in accordance with the Preferred Reporting Items for Systematic Review and Meta-Analysis statement (PRISMA) [9]. The following databases were fully searched from their inception date to January 2020: PubMed, EMBASE, the Cochrane Library, Web of Science^TM^, ClinicalTrials, ClinicalKey, and Google Scholar. For each database, a specific search strategy was developed using the following keywords: “Hip AND Fracture”, “Antiplatelet”, “Antithrombocyte”, “Platelet aggregation inhibitors”, “Aspirin”, “Plavix”, and “Clopidogrel” (detailed search strategies as shown in Additional file). Searches were without date or geographic restriction but were limited to primary studies written in English. All references of retrieved articles were also checked for additional relevant studies.

### Inclusion and exclusion criteria

Studies were included according to the following inclusion criteria: (1) randomized-controlled trials or high-quality observational studies; (2) studies that compared the use of antiplatelet drugs on admission with placebo or no treatment in hip fracture patients undergoing early surgery (the time from admission to theater < 5 days); and (3) studies that compared early surgery (< 5 days) with delayed surgery (> 5 days) for patients suffering hip fractures while on antiplatelet therapy. Based on previous studies and half-life of antiplatelet drugs, we set 5 days as the dividing line. The exclusion criteria were as follows: (1) studies comparing non-antiplatelet medication, such as warfarin or low molecular weight heparin; (2) non-clinical studies such as basic science studies, narrative reviews, surveys, letters, editorials, case series, case reports, comments, conference abstracts, or expert opinions; and (3) non-English studies. The potential overlap of subjects was evaluated by comparing demographic characteristics when multiple studies were conducted by the same author or research institute. Titles and abstracts were filtered and evaluated independently in a non-blinded standardized pattern. A final decision was made based on the adherence to the inclusion and exclusion criteria. Divergence was resolved by consensus.

**Fig. 1:**
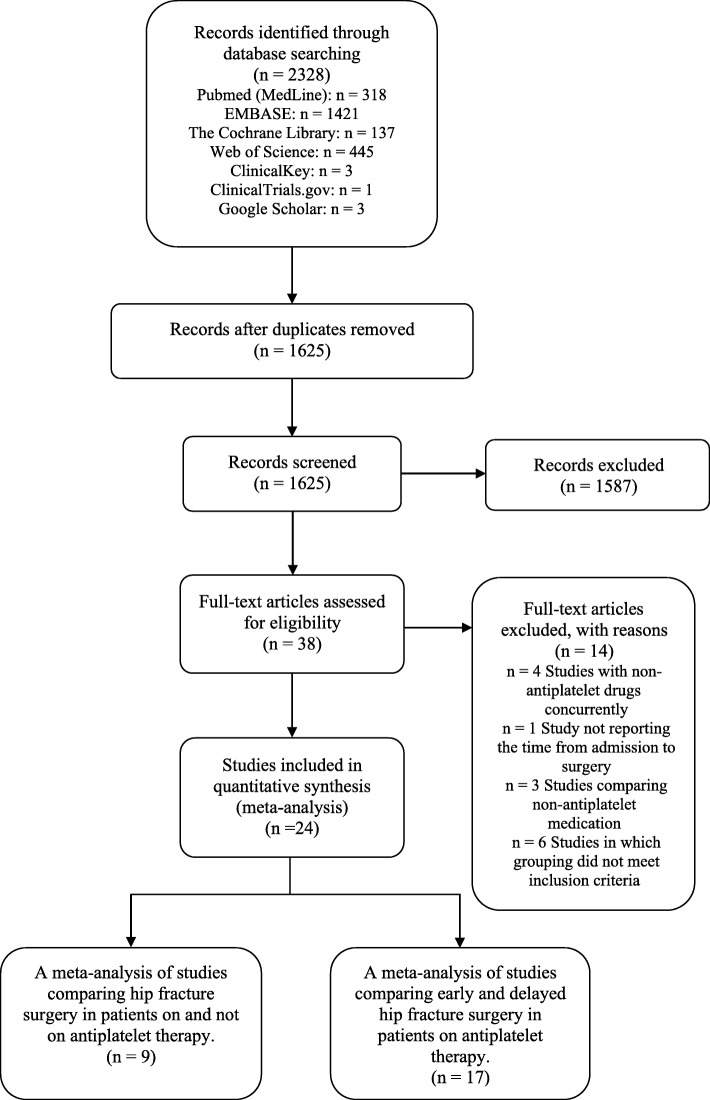
The flowchart of the study selection procedure is presented

As shown in Fig. 1, from the search, 2328 potentially eligible records were identified (including duplicates), 137 studies from The Cochrane Library, 318 studies from PubMed, 1421 studies from EMBASE, 445 studies from Web of Science^TM^, 1 study from ClinicalTrials, 3 studies from ClinicalKey, and 3 studies from Google Scholar. Removal of duplicates left 1625 articles. Of those studies, 1587 were excluded after their titles and abstracts were screened. The remaining 38 studies were read in full for eligibility. No additional studies were found from the references of the retrieved studies. Fourteen studies were eliminated for the following reasons: 4 studies with concurrent treatment with non-antiplatelet drugs; 1 study not reporting the time from admission to surgery; 3 studies comparing non-antiplatelet medication; and 6 studies in which grouping did not meet the inclusion criteria. The reasons for exclusion are listed in Table 1. Eventually, 24 studies were included in this review.

**Table 1:** Excluded studies and reasons for exclusion

Excluded studies	Reasons for exclusion
Manning et al. [10]	This study did not report the time from admission to theater.
Harty et al. [11]	Antiplatelet intervention group was with delayed surgery for more than 5 days.
Dettoni et al. [12]	Both antiplatelet intervention group and control group were discontinued for more than 5 days before surgery.
Leonidou et al. [13]	Antiplatelet intervention group was with delayed surgery for more than 5 days.
Nwachuku et al. [14]	These groups were divided into early surgery group or delayed surgery group based on the time from admission to theater less than or more than 48 h.
Lee et al. [15]	The study compared non-antiplatelet medication.
Drescher et al. [16]	The study compared non-antiplatelet medication.
Kulachote et al. [17]	The intervention group was with non-antiplatelet drugs concurrently.
Akaoka et al. [18]	The intervention group was with non-antiplatelet drugs concurrently.
Purushothaman et al. [19]	These groups were divided into early surgery group or delayed surgery group based on the time from admission to theater less than or more than 48 h.
Hwang et al. [20]	The intervention group was with non-antiplatelet drugs concurrently.
Zhang et al. [21]	Antiplatelet intervention group was with delayed surgery for more than 5 days.
Lott et al. [22]	The intervention group was with non-antiplatelet drugs concurrently.
Hoerlyck et al. [23]	The study compared non-antiplatelet medication.

### Assessment of study quality

No relevant randomized-controlled trials were detected; all of the included studies were comparative cohort studies in design. Therefore, the quality of the included studies was evaluated by the Newcastle/Ottawa scale (NOS) [24]. Two investigators independently scored items and assessed bias blinded to the study outcome. For each item, different response options were available, and a star system was adopted to provide a semi-quantitative evaluation of study quality. The NOS range is between zero and nine stars. Studies within 4 stars are considered to be of low quality, while those with 5 or more stars will be selected. The characteristics of all the included studies are presented in Table 2.

**Table 2:** Characteristics of included studies in the meta-analysis

Study	Country	Study type	Quality assessment	Duration	Sample size	Groups	Female (%)	Age (year)^a^	Time to surgery (day)^a^	Fracture type	Concurrent antiplatelet or thromboprophylaxis
Al Khudairy et al. [25]	Ireland	Cohort (retrospective)	**** * ***	24 months	47	Early VS delayed	66%	80.3 (8.3)	4.2 (1.0) VS 8.0 (1.0)	Intra and extracapsular	Unclear
Chechik et al. [26]	Israel	Quasi-randomized (retrospective)	**** * ***	34 months	60	Early VS delayed	50%	82.5 (7.9)	1.67 (1.0) VS 7.5 (2.70)	Intra and extracapsular	33% in early group and 17% in delayed group on aspirin 40 mg LMWH for thromboprophylaxis
Cox et al. [27]	UK	Cohort (retrospective)	**** _ **	2 years	20	Early VS delayed	65%	80.9	1.1 VS 7	Intra and extracapsular	Chemical or mechanical thromboprophylaxis
Johansen et al. [28]	UK	Cohort (retrospective)	**** _ **	18 months	17	Early VS delayed	Unclear	Unclear	2.7 VS 7.3	Unclear	Unclear
Pailleret et al. [29]	France	Cohort (retrospective)	**** ** **	6 years	39	Early VS delayed	77%	86 (7.5)	1 (1) VS 5 (1)	Intra and extracapsular	24% in early group and 32% in delayed group on aspirin 40 mg LMWH for thromboprophylaxis
Sa-Ngasoongsong et al. [30]	Thailand	Cohort (prospective and retrospective)	**** * ***	3 years	94	Early VS delayed	73%	80.5 (8.0)	1.6 (0.9) VS 8.9 (3.6)	Intra and extracapsular	42 aspirin and 7 clopidogrel in early group, 36 aspirin, and 9 clopidogrel in delayed group
Yoo et al. [31]	Korea	Cohort (retrospective)	**** * ***	3 years	43	Early VS delayed	70%	73.0 (7.1)	< 1 days VS 5.7	Intracapsular	Mechanical thromboprophylaxis was used and chemical prophylactic agents were not
Sim et al. [32]	Australia	Cohort (retrospective)	**** * ***	44 months	135	1) Early VS delayed 2) Antiplatelet VS no antiplatelet	76%	80.7 (9.7)	1) < 5 days VS > 5 days 2) 3.5 (3.2) VS 0.9 (0.8)	Intra and extracapsular	Unclear
Zehir et al. [33]	Turkey	Cohort (retrospective)	**** * ***	6 years	211	1) Early VS delayed 2) Antiplatelet VS no antiplatelet	55%	77.5 (7.6)	1) 1.79 VS 5.82 2) 1.79 VS 1.68	Intracapsular	40 mg LMWH for thromboprophylaxis
Anekstein et al. [34]	Israel	Cohort (prospective)	**** ** ***	14 months	104	Antiplatelet VS no antiplatelet	Unclear	77.1 (10.1)	1.53 (0.9) VS 1.48 (0.9)	Intra and extracapsular	Unclear
Chechik et al. [35]	Israel	Cohort (prospective)	**** * ***	21 months	88	Antiplatelet VS no antiplatelet	66%	81.8 (7.4)	2.15 (1.4) VS 1.88 (1.1)	Intra and extracapsular	Unclear
Collinge et al. [36]	USA	Cohort (retrospective)	**** ** **	5 years	946	Antiplatelet VS no antiplatelet	72%	80.8 (8.7)	1.54 (1.0) VS 1.55 (0.9)	Intra and extracapsular	A prophylactic doses of enoxaparin (Lovenox) within 24 h after surgery
Feely et al. [37]	USA	Cohort (retrospective)	**** ** ***	14 years and 6 months	120	Antiplatelet VS no antiplatelet	55%	82.2 (8.4)	1.1 (0.7) VS 1.3 (1.3)	Intra and extracapsular	2 cohorts had similar percentages of patients concurrent on aspirin and chemical thromboprophylaxis
Ghanem et al. [38]	USA	Cohort (retrospective)	**** * **	8 years	623	Antiplatelet VS no antiplatelet	69%	83.1	1.7 VS 1.3	Intracapsular	48% with aspirin in clopidogrel group, 38% in control group. A prophylaxis of enoxaparin was 22% in clopidogrel group and 30% in control group
Ginsel et al. [39]	Australia	Cohort (retrospective)	**** * ***	1 year	300	Antiplatelet VS no antiplatelet	71%	81.6 (13.1)	1.76 VS 1.6	Intracapsular	Unclear
Kennedy et al. [40]	Ireland	Cohort (retrospective)	**** _ **	NR	98	Antiplatelet VS no antiplatelet	73%	81.9	All patients < 2 days	Intra and extracapsular	Unclear
Kragh et al. [41]	Sweden	Cohort (retrospective)	**** * **	2 years	255	Antiplatelet VS no antiplatelet	54%	82.4 (8.8)	0.84 (0.4) VS 0.8 (0.4)	Intra and extracapsular	40 mg enoxaparin for thromboprophylaxis, 47% with compression bandage in antiplatelet group and 43% in non-antiplatelet group
Thaler et al. [42]	Austria	Cohort (prospective)	**** * ***	27 months	462	Antiplatelet VS no antiplatelet	74%	78 (11)	1.29 (1.9) VS 1.3 (2.0)	Intra and extracapsular	14% of clopidogrel group and 22% control group on aspirin. 40 mg enoxaparin for thromboprophylaxis
Clareus et al. [43]	Sweden	Cohort (retrospective)	**** ** ***	3 years	112	Antiplatelet VS no antiplatelet	68%	84.7 (7.3)	1.67 (1.2) VS 0.88 (0.5)	Intra and extracapsular	Unclear
Hossain et al. [44]	UK	Cohort (retrospective)	**** ** **	2 years	102	Antiplatelet VS no antiplatelet	81%	83.0 (7.5)	All patients < 2 days	Intracapsular	32% of clopidogrel group and 44% control group on aspirin. 40 mg enoxaparin postoperatively for 6 weeks for thromboprophylaxis
Manaqibwala et al. [45]	USA	Cohort (retrospective)	**** ** **	7 years	162	Antiplatelet VS no antiplatelet	69%	84.1 (8.9)	2.3 (2.0) VS 1.9 (2.9)	Intracapsular	66.7% of clopidogrel group and 43.5% control group on aspirin 40 mg enoxaparin or 5000 units heparin postoperatively for thromboprophylaxis
Nydick et al. [46]	USA	Cohort (retrospective)	**** ** *	5 years	50	Antiplatelet VS no antiplatelet	Unclear	Unclear	1.81 VS 1.65	Intra and extracapsular	Unclear
Wallace et al. [47]	USA	Cohort (retrospective)	**** ** ***	Over 5 years	110	Antiplatelet VS no antiplatelet	73%	79.9 (9.1)	All patients < 2 days	Intra and extracapsular	Mechanical thromboprophylaxis was used and chemical prophylactic agents were not
Wordsworth et al. [48]	UK	Cohort (prospective)	**** * ***	6 years	1225	Antiplatelet VS no antiplatelet	72%	82.3 (9.4)	1.23 VS 1.20	Intra and extracapsular	36.7% of clopidogrel group and 20% control group on aspirin 40 mg enoxaparin postoperatively for 2-4 weeks for thromboprophylaxis

A star system is used to allow a semi-quantitative assessment of study quality by using Newcastle/Ottawa scale

LMWH = low molecular weight heparin

5 or more stars for selection, _ = zero score for this domain, * = 1 point within this domain

^a^Values are mean (standard deviation)

### Data collection and abstraction

Two researchers independently extracted the data, including the title, lead author, publication year, country, study design, trial duration, number of participants, participant characteristics (mean age, gender, and fracture type), time to surgery, cohorts compared, surgical treatment, concurrent antiplatelet use, and perioperative use of thromboprophylaxis. When the trials had more than 2 groups and allowed multiple comparisons, we only collected the relevant information and data reported in the original articles. The number of events was extracted for all dichotomous outcomes and means, and standard deviations (SDs) were extracted for all continuous outcomes. If these values were not available, they were indirectly derived from *p* values or confidence intervals, if possible.

Outcomes were defined as a direct or indirect reflection of the surgical risk and prognosis of patients. All outcome data were extracted from included studies as far as possible. These included (1) in-hospital, 30-day, 3-month, and 1-year mortality; (2) blood transfusion exposures; (3) the average blood transfusion unit per patient; (4) decreases in hemoglobin; (5) length of hospital stay; (6) reoperation rate; and (7) postoperative complications including acute coronary syndrome, cerebrovascular events, deep vein thrombosis, pulmonary embolism, wound-related complications (infection and hematoma), and major bleeding (major bleeding was defined according to Eriksson et al. [49] as follows: (1) fatal bleeding, (2) excessive bleeding resulting in an intraoperative transfusion of four or more units of red blood cells, (3) bleeding involved any critical organ, and (4) bleeding that led to reoperation.

### Meta-analysis methodology

Actually, the following two meta-analyses were performed on the identified studies: (1) studies comparing early surgery (< 5 days) in hip fracture patients with antiplatelet therapy versus those without antiplatelet therapy and (2) studies comparing early surgery (< 5 days) versus delayed surgery (> 5 days) in patients with hip fractures receiving antiplatelet therapy. To evaluate whether there is a difference due to drugs between the antiplatelet and non-antiplatelet groups, we specified subgroups based on the antiplatelet treatment (aspirin, clopidogrel, or the combination of aspirin and clopidogrel). If possible, data were used from patients only on one specified drug while not on other antiplatelet drugs.

We performed a meta-analysis to calculate the odds ratios (ORs) or weighted mean differences (WMDs) presented with 95% confidence intervals (CIs) using the Mantel-Haenszel statistical method. According to the Cochrane Handbook [50], trials with no events in either the intervention or control group were not included in the meta-analysis when ORs were calculated. The *I*^2^ statistic was used to estimate the statistical heterogeneity between statistical data. A random-effects model was adopted when the heterogeneity was significant (*p* < 0.05), and a fixed-effects model was used if heterogeneity was absent. Publication bias was evaluated using funnel plots. Sensitivity analysis was performed by excluding studies without controlling for confounding variables or studies with characteristics different from the others. All meta-analyses were conducted using Review Manager 5.3, and *p* < 0.05 was regarded as statistically significant.

## Results

### Can early surgery be safely implemented on hip fracture patients who are treated with antiplatelet therapy?

A total of 17 studies were included to compare early surgery for hip fracture patients treated with antiplatelet therapy with those without antiplatelet therapy. As shown in Table 3, no significant differences in in-hospital mortality, 30-day mortality, or 1-year mortality were observed. However, there was substantial heterogeneity (*p* = 0.007; *I*^2^ = 68%) and an asymmetric funnel plot for 1-year mortality, which may be due to the trial by Kragh et al. [41]; thus, a random-effects model was used for this meta-analysis. Sensitivity analysis revealed that there was no evidence of heterogeneity in the remaining studies (*p* = 0.45; *I*^2^ = 0%), and it did not change the overall results when this outlier study was removed.

**Table 3:** Outcomes of meta-analysis in early surgery for hip fracture patients with or without antiplatelet therapy and subgroup analysis based on different drug regimens

Outcomes	No. of trials	No. of participants		WMD or OR (95% CI)	Subtotal *p* value	Subtotal Heterogenicity (*I*^2^ = %)	*p* value between subgroup (*I*^2^ = %)	*p* value
		Antiplatelet	Control					
In-hospital mortality								
Aspirin	3	11/456	24/1128	1.11 (0.54-2.32)	0.77	0	0.81	0.59
Clopidogrel	4	2/106	26/1242	1.17 (0.34-3.99)	0.80	0		
Clopidogrel and aspirin	1	1/34	8/619	2.31 (0.28-19.05)	0.44	Not applicable		
30-day mortality								
Aspirin	2	25/371	42/756	1.22 (0.72-2.07)	0.47	89	0.64	0.56
Clopidogrel	5	8/182	121/2070	1.20 (0.55-2.60)	0.65	0		
Clopidogrel and aspirin	1	1/34	39/619	0.45 (0.06-3.38)	0.44	Not applicable		
1-year mortality								
Aspirin	2	85/371	131/756	1.91 (0.56-6.54)	0.30	90	0.55	0.43
Clopidogrel	3	29/110	488/1534	0.90 (0.49-1.64)	0.72	41		
Clopidogrel and aspirin	1	7/34	117/619	1.11 (0.47-2.62)	0.81	Not applicable		
Drop in hemoglobin								
Aspirin	4	450	879	0.12 (− 0.06-0.31)	0.18	0	0.88	0.08
Clopidogrel	6	201	1017	0.16 (− 0.10-0.42)	0.22	0		
Clopidogrel and aspirin	1	34	619	0.00 (− 0.58-0.58)	1.00	Not applicable		
Number of patients receiving blood transfusion								
Aspirin	4	253/450	465/879	1.16 (0.91-1.46)	0.23	0	0.39	0.03
Clopidogrel	10	118/361	875/2965	1.19 (0.90-1.59)	0.23	36		
Clopidogrel and aspirin	1	24/34	337/619	2.01 (0.94-4.27)	0.07	Not applicable		
Mean number of units of blood transfused								
Aspirin	5	530	1185	0.13 (− 0.13-0.40)	0.32	49	0.23	0.07
Clopidogrel	7	250	2487	0.15 (− 0.25-0.56)	0.46	71		
Clopidogrel and aspirin	2	49	641	0.69 (0.10-1.28)	0.02	0		
Length of hospital stay								
Aspirin	2	380	808	− 0.39 (− 0.83-0.06)	0.09	0	0.03	0.76
Clopidogrel	6	257	1203	0.58 (− 0.17-1.34)	0.13	0		
Clopidogrel and aspirin	2	49	641	0.97 (− 0.40-2.34)	0.17	0		
Reoperation								
Aspirin	2	3/223	3/304	1.35 (0.28-6.61)	0.71	0	0.93	0.29
Clopidogrel	6	10/216	33/1037	1.47 (0.70-3.09)	0.31	0		
Acute coronary syndrome								
Aspirin	1	8/118	5/137	1.92 (0.61-6.04)	0.26	Not applicable	0.52	0.004
Clopidogrel	6	14/240	19/984	2.27 (1.07-4.81)	0.03	0		
Clopidogrel and aspirin	1	3/15	0/22	12.60 (0.60-264.14)	0.10	Not applicable		
Cerebrovascular events								
Aspirin	2	2/140	1/159	1.64 (0.27-9.79)	0.59	39	0.76	0.53
Clopidogrel	3	2/84	4/249	1.77 (0.35-9.04)	0.49	0		
Clopidogrel and aspirin	1	0/15	1/22	0.46 (0.02-12.12)	0.64	Not applicable		
Deep venous thrombosis								
Aspirin	3	9/476	11/923	1.50 (0.58-3.84)	0.40	61	0.94	0.30
Clopidogrel	4	2/162	14/1435	1.60 (0.45-5.74)	0.47	0		
Clopidogrel and aspirin	1	0/34	9/619	0.93 (0.05-16.33)	0.96	Not applicable		
Pulmonary embolism								
Aspirin	2	2/358	4/786	1.06 (0.22-5.14)	0.95	0	0.79	0.54
Clopidogrel	6	2/251	14/1664	1.37 (0.44-4.23)	0.59	0		
Clopidogrel and aspirin	1	0/34	2/619	3.58 (0.17-76.02)	0.41	Not applicable		
Wound-related complications								
Aspirin	4	14/498	25/945	0.86 (0.44-1.69)	0.67	0	0.34	0.48
Clopidogrel	8	14/287	35/2398	1.52 (0.75-3.09)	0.24	0		
Clopidogrel and aspirin	2	2/49	10/641	2.60 (0.49-13.74)	0.26	0		
Major bleeding								
Aspirin	1	3/98	10/342	1.05 (0.28-3.89)	0.94	Not applicable	0.58	0.48
Clopidogrel	3	5/91	13/444	1.75 (0.52-5.91)	0.37	0		

We found that antiplatelet therapy was significantly associated with an increase in the number of transfused patients (OR = 1.21; 95% CI, 1.01-1.44; *p* = 0.03). No evidence of statistical heterogeneity or publication bias was detected. Although the analysis of the three subgroups showed no differences in the transfusion rate, we focused on the overall results rather than on a separate subgroup because a test for interaction yielded a *p* value of 0.39.

There were no significant differences in the decline in hemoglobin or mean number of units of blood transfused between the two groups despite the increase in the transfusion rate. Moderate statistical heterogeneity (*p* = 0.002; *I*^2^ = 61%) was observed for the mean number of units for transfusion, and a random-effect model was applied. Subgroup analysis showed that the combination of aspirin and clopidogrel may result in an increase in the mean number of units for transfusion (WMD = 0.69; 95% CI, 0.10-1.28; *p* = 0.02). Sensitivity analysis was performed by excluding Zehir et al. [33], which was the primary source of statistical heterogeneity. This may be because in this study, the preoperative hemoglobin levels of the antiplatelet group were significantly lower than that of the control group. Following removal of this study, a remarkable decrease in heterogeneity (*p* = 0.08; *I*^2^ = 37%) was observed and the overall results remained unchanged.

There was also no significant difference in the length of hospital stay, reoperation, cerebrovascular events, deep vein thrombosis, pulmonary embolism, major bleeding, or other wound-related complications between the antiplatelet and non-antiplatelet groups, except for acute coronary syndrome (OR = 2.41; 95% CI, 1.32-4.42; *p* = 0.004). Subgroup analysis suggested that the results did not change due to treatment with aspirin, clopidogrel, or a combination of aspirin and clopidogrel. None of them showed significant heterogeneity or publication bias.

### Which is better, early or delayed surgery on hip fracture patients with antiplatelet therapy?

**Fig. 2:**
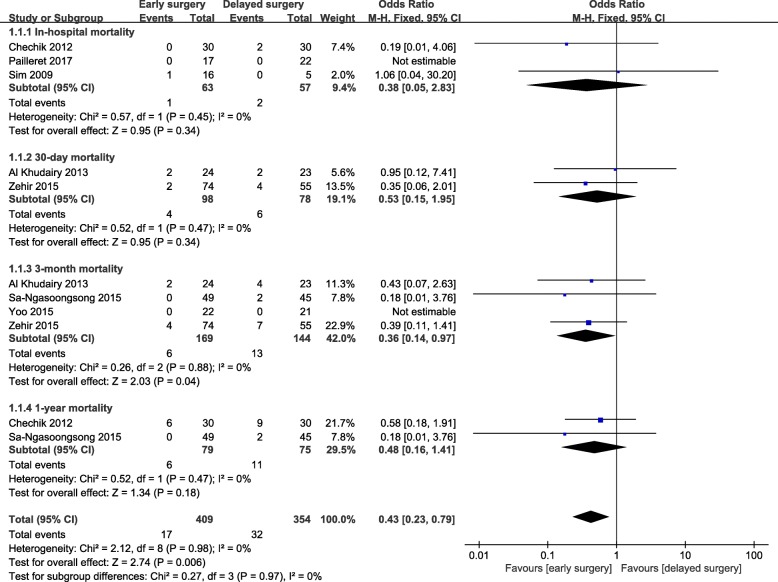
Meta-analysis of early surgery group and delayed surgery group: mortality

A total of 9 studies were included to compare early surgery (< 5 days) with delayed surgery (> 5 days) for hip fracture patients treated with antiplatelet therapy upon admission. There was a significant decrease in mortality (OR = 0.43; 95% CI, 0.23-0.79; *p* = 0.006) for those treated with antiplatelet therapy with early surgery (Fig. 2). No evidence of statistical heterogeneity or publication bias was observed. Sensitivity analysis did not change the overall results. Subgroup analysis revealed that the point estimate regarding the association of delayed surgery and mortality at any time point was increased, but only 3-month mortality reached statistical significance.

**Fig. 3:**
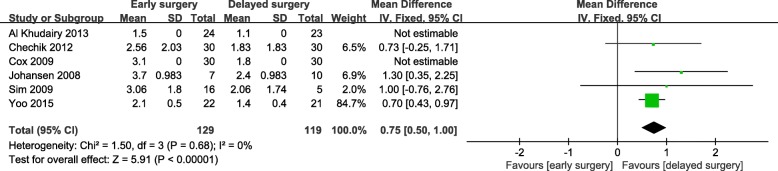
Meta-analysis of early surgery group and delayed surgery group: decrease in hemoglobin concentration

**Fig. 4:**
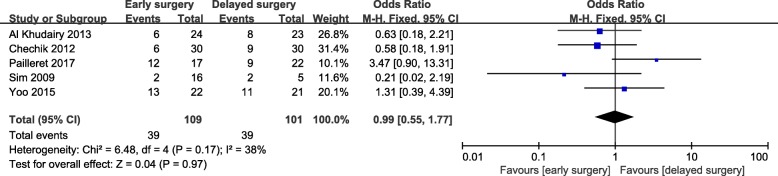
Meta-analysis of early surgery group and delayed surgery group: transfusion exposures

**Fig. 5:**
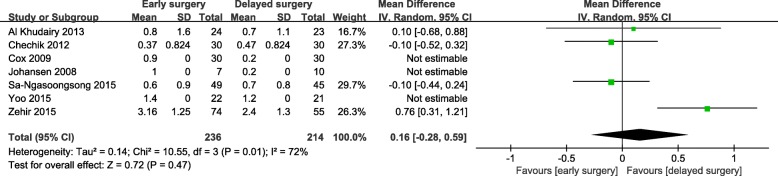
Meta-analysis of early surgery group and delayed surgery group: mean number of units of blood transfused

Early surgery was significantly associated with increased hemoglobin loss in hip fracture patients who received antiplatelet drugs (WMD = 0.75; 95% CI, 0.50-1.00; *p* < 0.001) (Fig. 3). However, there were no significant differences in the number of blood transfusions (Fig. 4) or mean number of units for transfusion (Fig. 5). There was a remarkable statistical heterogeneity (*p* = 0.01; *I*^2^ = 72%) and possible publication bias for the mean number of units for transfusion. Sensitivity analysis was performed by separately excluding Zehir et al. [33], and the results remained unchanged.

**Fig. 6:**
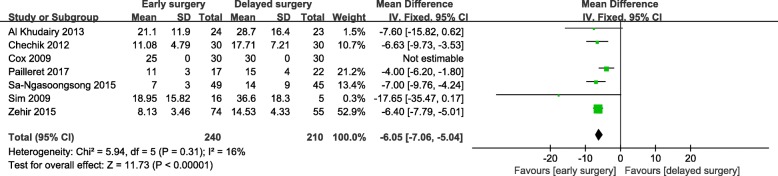
Meta-analysis of early surgery group and delayed surgery group: length of hospital stay

**Fig. 7:**
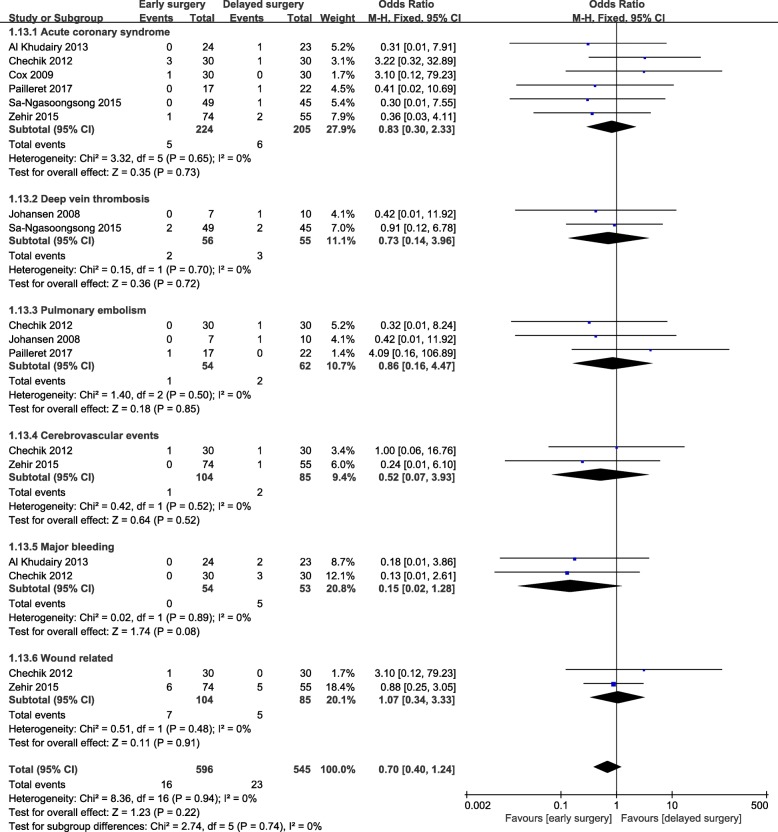
Meta-analysis of early surgery group and delayed surgery group: postoperative complications

The length of hospital stay in the early surgery group was significantly shortened (WMD = − 6.05; 95% CI, − 7.06-5.04; *p* < 0.001) (Fig. 6). Nevertheless, there were no significant differences for acute coronary syndrome, cerebrovascular events, deep vein thrombosis, pulmonary embolism, major bleeding, or other wound-related complications (Fig. 7). Moreover, no heterogeneity was observed, and the results were not altered by separately excluding subgroups after sensitivity analysis.

## Discussion

Hip fracture patients tend to be older, with a high mortality rate, and their quality of life is seriously affected [51]. As the global elderly population is gradually increasing, the number of elderly patients with hip fractures and the socioeconomic burden are also increasing year by year [52, 53]. Most of them usually suffer from cardio-cerebrovascular disease and need antiplatelet therapy. Some believe that it is necessary to withhold antiplatelet therapy to promote platelet function recovery and reduce the risk of perioperative bleeding. Others believe that surgery should be performed as soon as possible without stopping medication. Previous literature has found that there is an absence of a consensus or policies for the treatment of patients who sustain hip fractures while on antiplatelet therapy. A telephone questionnaire data analysis about current practical measures among 110 orthopedics in the UK showed that 56.4% of orthopedics did not have a standard of clopidogrel withdrawal, and the remaining 43.6% stopped clopidogrel before surgery. Among them, 20.9% of the surgeries were delayed by more than 5 days, and 12.7% were delayed by 7–10 days [54].

Although Soo et al. [55] and Doleman et al. [56] tried to identify how to manage these cases, the total number of studies included in these reviews were low, and these studies may result in type II errors and were either limited to one specific antiplatelet drug or confused the presence or absence of antiplatelet drugs with early or delayed surgery. Thus, we performed a more systematic and specific search and analysis to address the issues highlighted from previous research. The important finding of our study is that early surgery for hip fracture patients taking antiplatelet drugs might promote a higher risk of bleeding and more blood transfusion requirements compared to those without antiplatelet therapy. Nevertheless, there were no significant differences in prognosis. Instead, delayed intervention will lead to higher mortality and a longer hospital stay.

There were several limitations to our study. One of the limitations was that the methodological quality of the studies included was not optimal. Only observational studies were included in our analysis, which means that only the inference of association is possible rather than causality; there may be potential confounding variables that bias the outcomes. For instance, there were three main types of hip fracture surgery in included studies: hip repair using internal fixation, partial hip replacement surgery, and total hip replacement surgery. Different surgical methods will affect the outcomes, but most of the included studies did not distinguish and explain so that we were unable to exclude this confounding factor. As expected, the intervention groups in most studies [37, 38, 40, 41, 44, 45, 47] showed a significant increase in the number of cardiovascular or cerebrovascular comorbidities; however, surprisingly, only three of them showed a significant difference in the ASA grade [38, 41, 45]. Moreover, the preoperative hemoglobin values of the intervention group in five studies [30, 33, 45, 46] were significantly lower than those of the control group, which may potentially influence blood transfusions, meaning that the intervention groups required more units of blood. This may be why Zehir et al. [33] was the main source of heterogeneity in the outcomes for the mean number of units for transfusion. A further limitation was that publication bias existed in some studies as shown in the funnel plots; this might be because the number of included trials was less than 10. Finally, although we performed subgroup analysis based on the types of antiplatelet drugs and data were used from patients on one specific drug and not on the others simultaneously as much as possible, most of the trials included patients concurrently treated with aspirin in the clopidogrel subgroup, and this may affect the final results.

Regarding whether early surgery is safe for hip fracture patients taking antiplatelet drugs, the number of patients transfused in the antiplatelet group increased statistically, which was consistent with that in cardiac surgery [57, 58]. However, we found no convincing evidence of an increase in the average blood transfusion demands, except for in the medicine-united group. This suggested that there might indeed be an increased risk of bleeding in intraoperative blood loss or hidden blood loss, especially when antiplatelet drugs are used in combination [35]. However, because of the concerns of antiplatelets from anaesthesiologists and physicians, the patients taking antiplatelet drugs are more likely to have a lower threshold to receive transfusions. No differences in mortality, duration of hospital stay, reoperation rate, or related complications, except acute coronary syndrome, were detected between the two groups. The presence of more vascular comorbidities in the antiplatelet group of most studies may be responsible for the significant increase in acute coronary syndrome.

Regarding whether early or delayed surgery is better for patients with hip fractures on antiplatelet therapy, early surgery was associated with a greater decrease in hemoglobin; however, there were no differences in the transfusion rate or mean number of units for transfusion. This also supports the fact that patients taking antiplatelet drugs are more likely to be transfused owing to potential performance bias. Multiple studies have shown that delays in surgery for more than 2 days for hip fracture patients are closely related to an increased risk of complications due to long-term bedridden and delayed mobilization [59, 60]. Early surgical intervention can significantly reduce postoperative mortality and morbidity, promote a shorter hospital stay, and prompt patients to return to pre­injury ambulation status [61–65]. However, early surgery for patients on antiplatelet may cause hemorrhagic accidents, as platelet function has not fully recovered [66]. In our study, delayed surgery increased the risk of mortality, and subgroup analysis showed that the point estimate regarding mortality at any time point was increased, especially mortality at 30 days and 3 months, which showed significant differences. Furthermore, hip fractures are more likely to prolong the length of hospital stay than any other musculoskeletal injuries, accounting for more than two-thirds of all hospital stays caused by fractures [67]. Early surgery can effectively shorten the length of hospital stay and reduce social and economic burdens. Unlike previous research studies, our study suggests that there are no differences in the incidence of postoperative complications between early and delayed surgery. Previous studies have demonstrated that sudden withdrawal will lead to conversion to a prothrombotic and proinflammatory condition, which may complicate surgery and lead to adverse clinical events, such as recurrence and death by myocardial infarction, which has already been stabilized by drugs or stents [68]. However, in the meta-analysis reported here, subgroup analysis showed that a surgical delay did not have a higher postoperative incidence of cardiocerebrovascular events or thromboembolic events, and early surgery did not result in a higher incidence of severe bleeding.

## Conclusion

In conclusion, our analysis of 24 trials including a total of 5423 patients suggests that early surgery can be safely performed on hip fracture patients receiving antiplatelet drugs upon admission. Current evidence shows that although early surgery carries a high risk of bleeding, it does not lead to substantial blood transfusion demands or hemorrhagic events. Furthermore, compared with delayed surgery, early surgical intervention is associated with a significant decrease in mortality (*p* = 0.006) and length of hospital stay (*p* < 0.001). Based on the available evidence, it is unnecessary to delay surgery to restore platelet function when patients with hip fractures receive antiplatelet therapy. Early surgery can significantly reduce mortality and hospital stay, which is conducive to patient recovery. Further large-scale, multi-centered, well-motivated and well-designed randomized trials are required to confirm these findings and develop clearer guidelines for the treatment of these patients.

## Supplementary information

**Additional file 1.**

## Data Availability

All data generated or analyzed during this study are included in published articles.

## Abbreviations

PRISMA: Preferred Reporting Items for Systematic Review and Meta-Analysis statement
NOS: Newcastle/Ottawa scale
SDs: Standard deviations
ORs: Odds ratios
WMDs: Weighted mean differences
CIs: Confidence intervals
