# Supplementary material for: Is hip fracture surgery safe for patients on antiplatelet drugs and is it necessary to delay surgery? A systematic review and meta-analysis
Source: J Orthop Surg Res. 2020 Mar 12;15:105. doi: 10.1186/s13018-020-01624-7 (PMC7068917; doi:10.1186/s13018-020-01624-7)
Supplement: Supplementary file 1 — Additional file 1. [file 13018_2020_1624_MOESM1_ESM.docx]

**Additional files 1**

**1.Pubmed**

#1 “hip”[MeSH Terms] OR hip*[All Fields]

#2 “femur”[MeSH Terms] OR femur*[All Fields] OR femoral*[All Fields] OR trochant*[All Fields] OR intertrochant*[All Fields] OR subtrochant*[All Fields] OR pertrochant*[All Fields]

#3 “fractures,bone”[MeSH Terms] OR fracture*[All Fields]

#4 #1 OR #2

#5 #3 AND #4

#6 “platelet aggregation inhibitors”[MeSH Terms] OR platelet aggregation inhibitors [All Fields] OR thrombocyte aggregation inhibitors[All Fields]

#7 Platelet* [All Fields] OR thrombocyt*[All Fields]

#8 antiaggregant* [All Fields] OR anti-aggregant* [All Fields] OR inhibit* [All Fields] OR antagonist* [All Fields]

#9 Antiplatelet* [All Fields] OR anti-platelet* [All Fields]

#10 Antithrombocyt* [All Fields] OR anti-thrombocyt* [All Fields]

#11 agent* [All Fields] OR drug*[All Fields] OR therap*[All Fields] OR regime*[All Fields] OR treatment*[All Fields] OR intervention*[All Fields]

#12 “aspirin”[MeSH Terms] OR aspirin*[All Fields] OR acetylsalicylic acid[All Fields]

#13 “clopidogrel”[MeSh Terms] OR clopidogrel [All Fields] OR plavix [All Fields]

#14 #7 AND #8

#15 #9 AND #11

#16 #10 AND #11

#17 #6 OR #12 OR #13 OR #14 OR #15 OR #16

#18 #5 AND #17

**2.Embase**

#1 ‘hip’/exp OR hip*

#2 ‘femur’/exp OR femur* OR femoral* OR trochant* OR intertrochant* OR subtrochant* OR pertrochant*

#3 ‘fracture’/exp OR fracture*

#4 #1 OR #2

#5 #3 AND #4

#6 'antithrombocytic agent'/exp

#7 platelet* OR thrombocyt*

#8 antiaggregant* OR 'anti aggregant*' OR inhibit* OR antagonist*

#9 antiplatelet* OR 'anti platelet*'

#10 antithrombocyt* OR 'anti thrombocyt*'

#11 agent* OR drug* OR therap* OR regime* OR treatment* OR intervention*

#12 'acetylsalicylic acid'/exp

#13 aspirin* OR 'acetylsalicylic acid' OR clopidogrel OR Plavix

#14 #7 AND #8

#15 #9 AND #11

#16 #10 AND #11

#17 #6 OR #12 OR #13 OR #14 OR #15 OR #16

#18 #5 AND #17

**3.The Cochrane Library**

#1 MeSH descriptor: [Hip] explode all trees

#2 hip*:ti,ab,kw (Word variations have been searched)

#3 MeSH descriptor: [Femur] explode all trees

#4 femur*:ti,ab,kw OR femoral*:ti,ab,kw OR trochant*:ti,ab,kw OR intertrochant*:ti,ab,kw OR subtrochant*:ti,ab,kw OR pertrochant*:ti,ab,kw (Word variations have been searched)

#5 MeSH descriptor: [Fractures, Bone] explode all trees

#6 fracture*:ti,ab,kw (Word variations have been searched)

#7 #1 OR #2 OR #3 OR #4

#8 #5 OR #6

#9 #7 AND #8

#10 MeSH descriptor: [Platelet Aggregation Inhibitors] explode all trees

#11 Platelet* OR thrombocyte*:ti,ab,kw (Word variations have been searched)

#12 antiaggregant* OR anti-aggregant* OR inhibit* OR antagonist*:ti,ab,kw (Word variations have been searched)

#13 antiplatelet* OR anti-platelet*:ti,ab,kw (Word variations have been searched)

#14 antithrombocyt* OR anti-thrombocyt*:ti,ab,kw (Word variations have been searched)

#15 agent* OR drug* OR therap* OR regime* OR treatment* OR intervention*:ti,ab,kw (Word variations have been searched)

#16 MeSH descriptor: [Aspirin] explode all trees

#17 aspirin* OR acetylsalicylic acid:ti,ab,kw (Word variations have been searched)

#18 clopidogrel OR plavix:ti,ab,kw (Word variations have been searched)

#19 #11 AND #12

#20 #13 AND #15

#21 #14 AND #15

#22 #10 OR #16 OR #17 OR #18 OR #19 OR #20 OR #21

#23 #9 AND #22

**4.Web of Science**

#1 TS=(hip* OR femur* OR femoral* OR trochant* OR intertrochant* OR subtrochant* OR pertrochant*)

#2 TS=(fracture*)

#3 #1 AND #2

#4 TS=(Platelet Aggregation Inhibitors OR thrombocyte aggregation inhibitors)

#5 TS=(Platelet* OR thrombocyt*)

#6 TS=(antiaggregant* OR anti-aggregant* OR inhibit* OR antagonist*)

#7 TS=(Antiplatelet* OR anti-platelet*)

#8 TS=(Antithrombocyt* OR anti-thrombocyt*)

#9 TS=(agent* OR drug* OR therap* OR regime* OR treatment* OR intervention*)

#10 TS=(aspirin* OR acetylsalicylic acid OR clopidogrel OR plavix)

#11 #5 AND #6

#12 #7 AND #9

#13 #8 AND #9

#14 #4 OR #10 OR #11 OR #12 OR #13

#15 #3 AND #14
